# Health-related quality of life in men with prostate cancer undergoing active surveillance versus radical prostatectomy, external-beam radiotherapy, prostate brachytherapy and reference population: a cross-sectional study

**DOI:** 10.1186/s12955-019-1082-4

**Published:** 2019-01-14

**Authors:** A. Sureda, L. Fumadó, M. Ferrer, O. Garín, X. Bonet, M. Castells, M. C. Mir, J. M. Abascal, F. Vigués, L. Cecchini, J. F. Suárez

**Affiliations:** 1 Urology Department, Hospital del Mar-IMIM, Autonomous University of Barcelona, Passeig Marítim 25-29, 08003 Barcelona, Spain; 2 Autonomous University of Barcelona, Barcelona, Spain; 3 IMIM (Institut Hospital del Mar d’Investigacions Mèdiques), Barcelona, Spain; 4 CIBER en Epidemiología y Salud Pública, CIBERESP, Barcelona, Spain; 5 Universitat Pompeu Fabra, Barcelona, Spain; 6 Hospital Universitari de Bellvitge, l’Hospitalet de Llobregat, Barcelona, Spain

**Keywords:** Active surveillance, Brachytherapy, External-beam radiotherapy, Health-related quality of life, Radical prostatectomy

## Abstract

**Background:**

The purpose of this study is to describe Health-Related Quality of Life (HRQoL) of localized prostate cancer patients in an Active Surveillance (AS) program, and to compare them with those undergoing radical prostatectomy (RP), external-beam radiotherapy (XRT) and brachytherapy (BT).

**Methods:**

Multi-institutional pooled cross-sectional analysis on patients in an AS protocol: < 75 years old; pathologically confirmed LPC (maximum of three positive cylinders); Gleason score < 3 + 4; clinical stage T1a-T2b; and PSA < 15 ng/ml. Exclusion criteria for this study were: less than 6 months in AS, termination of AS protocol, or incomplete data. Patients in AS were matched with those treated with RP, XRT or BT from the ‘Spanish Multicentric Study of Clinically Localized Prostate Cancer’ cohort according to risk group, time from treatment selection to HRQoL survey, and age. Prostate-specific (EPIC) and generic (SF-36) HRQoL instruments were completed. Analysis was stratified by HRQoL survey moment (>or < 2.5 years from treatment selection), and age (>or < 70 years old).

**Results:**

Median of time from treatment selection to HRQoL survey in the total 396 patients (99 per treatment group) was 2.4 years (range 0.5–8.3). Patients in AS presented higher (better) urinary incontinence scores than RP ones in both stratus of time from treatment selection to HRQoL survey (92.6 vs 67.0 and 81.4 vs 64.4, *p* <  0.01). Patients in AS for < 2.5 years presented greater sexual scores than any active treatment (p <  0.01), but only statistically higher than RP for those in AS for longer than 2.5 years. The magnitude of the differences between AS and RP groups in both EPIC domains ranged from moderate (0.7 SD) to large (1.0 SD).

Regardless of treatment applied, patients presented similar and slightly increased SF-36 scores than US general population reference norms. Nonetheless, patients in AS for < 2.5 years reported worse outcomes than other treatment groups on physical health domains, especially in bodily pain (0.5–0.6 SD), and vitality (0.6–0.8 SD).

**Conclusions:**

Considering patients’ well-being, AS can be a good therapeutic option due to the low impact caused on urinary continence and sexual function. However, longitudinal studies are required to take into account HRQoL evolution over time.

**Electronic supplementary material:**

The online version of this article (10.1186/s12955-019-1082-4) contains supplementary material, which is available to authorized users.

## Background

Prostate cancer (PC) is overdiagnosed and overtreated due to the increased utilization of prostate-specific antigen (PSA) [1]. The PIVOT trial demonstrated no decrease in prostate cancer mortality with the use of surgical treatment, compared to observation, for localized PC, except for the high-risk population [2]. Available curative treatments for localized PC [radical prostatectomy(RP), external-beam radiotherapy(XRT) and brachytherapy(BT)] may decrease the patient’s health-related quality of life (HRQoL) [3, 4]. Active Surveillance(AS) has been postulated as a safe alternative for patients with favourable-risk PC in order to reduce treatment side effects, with no impact on short-term survival. Several published series have demonstrated its validity reducing overtreatment [5–8].

Although living with cancer may cause psychological disorders, several studies proved that the impact of AS on patient’s mental health is low or null [9–11]. Previous studies [12–18] have evaluated the impact of AS using generic HRQoL measures, with results consistently demonstrating minimal declines in comparison to active treatments. The English ProtecT study, the first randomized clinical trial comparing treatments for localized prostate cancer in the PSA era, showed that patients allocated to the arm of AS presented similar sexual dysfunction and sexual bother than those allocated to radiotherapy at the 3rd year of follow-up [4]. These results are consistent with findings from a previous population-based Australian prospective cohort study [19], and with a longitudinal study from USA [20]. However, another USA study showed significantly less sexual dysfunction among AS patients than those treated with radiotherapy at the 3rd year of follow-up [21]. It is important to highlight that, since these studies evaluated the treatments’ efficacy and effectiveness, the AS arm included also patients who underwent RP or XRT at some point during follow-up: 14% at the 2nd year in the Australian study [19]; 19% at the 2nd year [20] and 24% at the 3rd year [21] in the USA studies; and around 20, 40 and 55% at 2nd, 5th and 10th year, respectively, in the ProtecT trial [22]. This ‘intention to treat’ analytical strategy prevents a clear picture of patients who remained on AS without radical treatment.

The main purpose of this study was to describe HRQoL of patients with localized PC in an AS program, without any other treatment. In order to compare them with those undergoing RP, XRT and BT, data from the ‘Spanish Multicentric Study of Clinically Localized Prostate Cancer’ [23, 24] was used. Physical and mental health components were also compared to general population [25, 26]. Our hypothesis are that: 1) HRQoL of localized PC patients in an AS program is better than in those treated by available curative treatments, since they presented less side effects; and 2) Patients in AS have poorer mental health, in comparison to those undergoing available curative treatments or general population, due to the uncertainty regarding their disease’s progression.

## Methods

Cross-sectional study of patients undergoing AS in two hospitals, matched with patients treated with RP, XRT or BT from the ‘Spanish Multicentric Study of Clinically Localized Prostate Cancer’ [23, 24] cohort, according to risk group, elapsed time from treatment selection to HRQoL survey (+/− 1 year), and age (+/− 5 years old).

### Subjects

Patients who entered in the AS protocol at the host institutions (Hospital Universitari de Bellvitge - l’Hospitalet de Llobregat, Spain - and Hospital del Mar - Barcelona, Spain) with primary diagnosis of PC between January 2008 and June 2015, were included (*n* = 180). The institutional review board at each institution approved the AS protocols independently, and written consent from the participants was obtained. AS protocol criteria were: < 75 years old; pathologically confirmed PC (maximum of three positive cylinders); Gleason score < 3 + 4; clinical stage T1a-T2b; and PSA < 15 ng/ml. Exclusion criteria for this study were: less than 6 months in AS, termination of AS protocol, incomplete data, or refusal to participate. Risk stratification was performed according to d’Amico criteria [27].

**Fig. 1:**
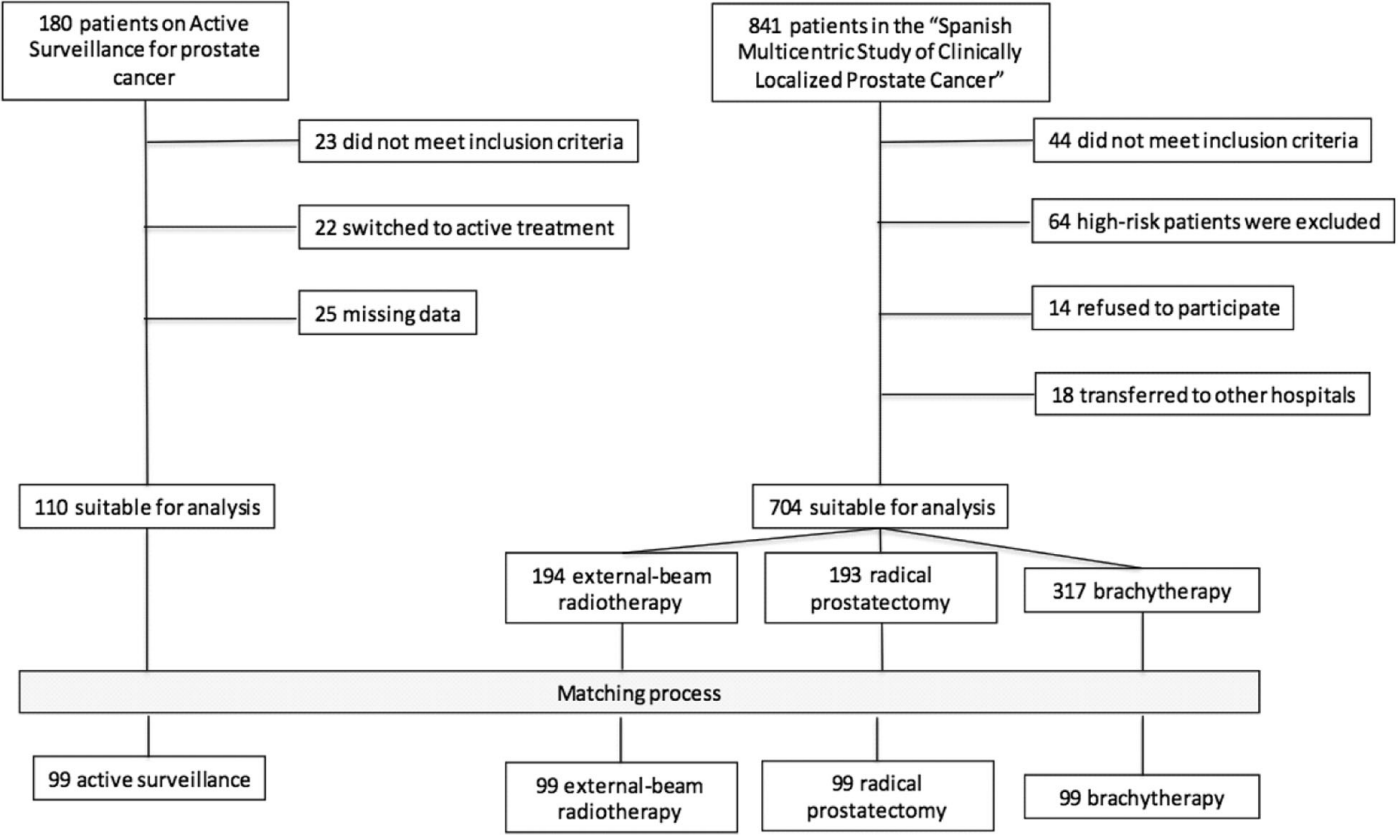
Study flowchart of enrolment and exclusion criteria that was used to select the cases suitable for the analysis

Demographic and clinical baseline characteristics were collected at the pre-assessment visit (age, PSA level, clinical stage, Gleason grade). From a total of 180 patients registered in the AS program: 23 were excluded because they did not meet protocol criteria, 22 because they had switched to active treatment due to clinical progression, and 25 due to missing data (Fig. 1). Finally, during 2016, the HRQoL survey was administered centrally by telephone interview (see below “HRQoL instruments”) to 110 patients at a median of 2.4 years after entering AS (range 0.5–8.3).

### ‘Spanish Multicentric Study of Clinically Localized Prostate Cancer’ cohort

Data for comparative active treatment groups were extracted from the “Spanish Multicentric Study of Clinically Localized Prostate Cancer”, which included patients treated with RP, XRT and BT, while not in AS since it was not a usual option in Spain when the study was designed in 2000. Details of this cohort have been described previously [23, 24]. Briefly, a total of 704 patients diagnosed of low/intermediate risk PC were recruited between 2003 and 2005. Patients were prospectively enrolled in the protocol and followed subsequently. Treatment was elected at patient-physician choice: 193 underwent RP, 194 XRT and 317 BT (Fig. 1). HRQoL data were collected by telephone interview before treatment and at one, three, six and twelve months post-treatment the first year, and annually thereafter.

Of the 704 patients, 26 had died at the third year after treatment - only 4 deaths related to PC. From the 678 alive, 500 completed the HRQoL assessment at three years after treatment (74% HRQoL completion rate), and only 87 (12.8%) missed the consecutive interviews (years 4 and 5). Those 500 patients were matched with the 110 in AS by randomized selection according to risk group, elapsed time from treatment selection to HRQoL survey (+/− 1 year), and age (+/− 5 years old).

Supplementary data related to non-matched patients is also described (See Additional file 1: Table S1).

### HRQoL instruments

A single trained nurse performed the telephone interviews and data collection, avoiding potential internal differences due to interviewer bias. Total telephone interview time took between 45 and 60 min.

The disease-specific instrument used to evaluate the impact of treatment side effects on HRQoL was the Expanded Prostate Cancer Index Composite (EPIC) [28]. It contains 50 items from five domains (urinary incontinence, urinary irritative-obstructive, bowel, sexual and hormonal). All EPIC items have a reminder period of four weeks, and are answered on a 5-point Likert scale. Scores are transformed linearly to scales from 0 to 100.

The 36-Item Short Form Health Survey (SF-36) version 2 [25, 29] contains 36 items covering 8 domains (physical functioning, role-physical, bodily pain, general health, vitality, social functioning, role-emotional and mental health). Two additional component summaries were generated (physical and mental). All domains and summary scores were obtained following the authors’ recommendations [29] by standardization to a mean of 50 and a standard deviation (SD) of 10 for the US general population. These standardized scores were developed to facilitate interpretability [30, 31] by allowing the direct comparison with the mean of the US general population, and an easy translation of score differences in terms of effect sizes given its SD = 10.

In both HRQoL instruments, EPIC and SF-36, higher scores imply better quality of life. The magnitude or clinical importance of score differences can be interpreted using the standard categorization of effect size [32], whereby 0.2, 0.5 and 0.8 of the SD represent small, moderate and large differences, respectively.

### Statistical analysis

Each of the 110 patients undergoing AS was matched to one patient from each attempted curative treatment group by randomized selection according to pre-defined attributes (risk group, elapsed time from treatment selection to HRQoL survey, and age), giving priority to patients with less potential pairs. The matching process allowed to pair 99 patients treated by AS with patients under each treatment group from the ‘Spanish Multicentric Study of Clinically Localized Prostate Cancer’ cohort (total *n* = 396). The statistical power was 80%, with a type I error of 5% to detect differences of twelve points (effect size of 0.5 SD) on the sexual score of EPIC questionnaire between groups with 50 patients.

To describe baseline characteristics, mean (SD) was calculated for quantitative variables or frequencies, and percentages for categorical variables. Comparisons among four treatment groups were tested using Chi squared test or one-way analysis of variance (ANOVA), depending on the variable’s nature. Post hoc comparisons to test differences between AS and any other treatment were performed by the Tukey Studentized Range (HSD) test for continuous variables or by Chi squared test for categorical variables.

Taking into account that HRQoL may differ according to the elapsed time from treatment selection to survey and age, we performed the analyses stratifying by them. In order to obtain two groups of similar size, the cut-off that approximated the median was selected for each of these two variables. Figures were constructed to show mean and 95% confidence interval of HRQoL scores by treatment group, and also the mean of men aged 65–74 years old in the general population reference norm of the SF-36.

## Results

Relevant baseline characteristics of the 396 patients (99 per treatment group) are described in Table 1. No differences were found among groups on the variables used in the matching process, except for age: patients in the AS group were older than RP ones (70.6 vs 68.2 year) and younger than XRT ones (70.6 vs 73.2 year). The median (range) of time elapsed from treatment selection to the HRQoL survey was: 2.4 (0.53–7.32) in the AS group, 3.1 (0.45–8.32) in the RP group, 2.0 (0.46–8.32) in the XRT group and 3.0 (0.48–7.06) in the BT group.

**Table 1:** Demographic and clinical baseline characteristics according to treatment group

	Active Surveillance (*n* = 99)	Radical prostatectomy (*n* = 99)	External-beam radiotherapy (*n* = 99)	Brachytherapy (*n* = 99)	*p*-value
Risk group*					
Low risk	74 (74.7%)	74 (74.7%)	74 (74.7%)	74 (74.7%)	1.000
Intermediate risk	25 (25.3%)	25 (25.3%)	25 (25.3%)	25 (25.3%)	
Age at HRQoL survey, mean (SD), years	70.6 (5.4)	68.2 (5.4)	73.2 (5.3)	71.9 (5.0)	0.065
Elapsed time from treatment selection to HRQoL survey, years					
Mean (SD)	2.6 (1.5)	3.1 (1.9)	2.8 (1.9)	2.9 (1.7)	0.434
Median [IQR]	2.4 [1.4–3.6]	3.1 [1.0–4.1]	2.0 [1.0–4.1]	3.0 [1.0–4.1]	
Minimum - Maximum	0.53–7.32	0.45–8.32	0.46–8.32	0.48–7.06	
PSA, mean (SD), ng/ml	6.8 (2.9)	7.3 (2.8)	7.7 (3.0)	7.6 (2.6)	0.087
PSA ≤ 10	84 (84.8%)	88 (89.8%)	84 (84.8%)	85 (85.9%)	0.709
PSA > 10	15 (15.2%)	10 (10.2%)	15 (15.2%)	14 (14.1%)	
Gleason score					
2–5	0	12 (12.1%)	30 (30.3%)	28 (28.2%)	< 0.001^a,b,c^
6	90 (90.9%)	72 (72.7%)	53 (53.5%)	64 (64.6%)	
7 (3 + 4)	9 (9.1%)	15 (15.2%)	16 (16.2%)	7 (7.1%)	
Clinical stage					
T1a-T1c	78 (78.8%)	71 (71.7%)	68 (68.7%)	80 (80.3%)	0.157
T2a-T2b	21 (21.2%)	28 (28.3%)	31 (31.3%)	19 (19.2%)	
Comorbidities					
Osteoarthritis or Rheumatism		31 (43.1%)	44 (48.9%)	40 (50.6%)	0.623
High blood pressure		26 (36.1%)	33 (36.7%)	33 (41.8%)	0.723
Depression or Mental disorders		13 (18.1%)	12 (13.3%)	17 (21.5%)	0.370
Chronic respiratory diseases		7 (9.7%)	17 (18.9%)	16 (20.3%)	0.168
Ischemic heart disease		6 (8.3%)	16 (17.8%)	11 (13.9%)	0.220
Diabetes mellitus		8 (11.1%)	13 (14.4%)	10 (12.7%)	0.818
Stroke		2 (2.8%)	3 (3.3%)	4 (5.1%)	0.737

*According to d’Amico prostate cancer risk classification

a: active surveillance vs radical prostatectomy; b: active surveillance vs external-beam radiotherapy; c: active surveillance vs brachytherapy

**Fig. 2:**
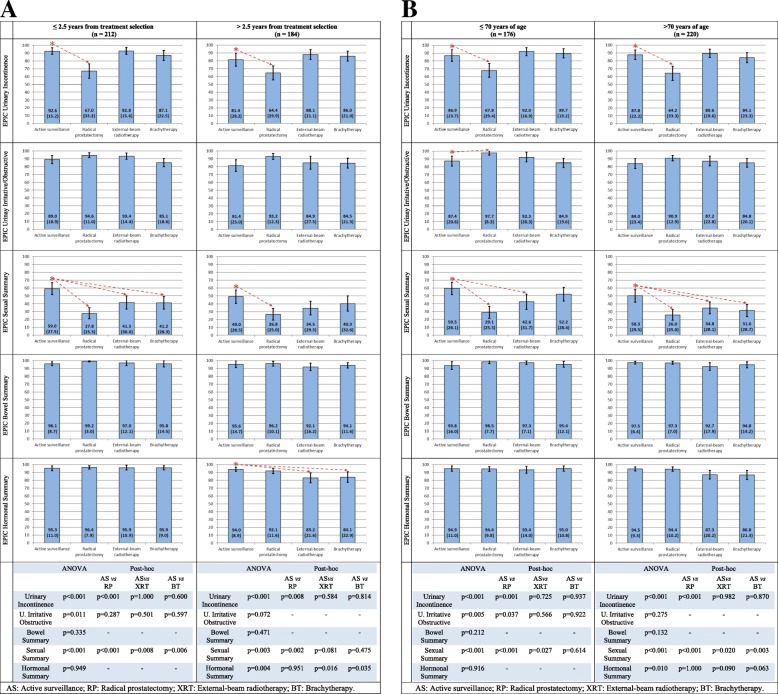
Health-related quality of life (HRQoL) in patients with localized prostate cancer, measured with EPIC. **a** Stratified by elapsed time from treatment selection to HRQoL survey. **b** Stratified by age at HRQoL survey

EPIC results are described in Fig. 2. AS patients reported higher urinary incontinence scores (higher scores mean better continence) related to RP in both stratus of time from treatment selection to HRQoL survey (92.6 vs 67.0 and 81.4 vs 64.4, *p* < 0.01). Patients in AS for 2.5 years or less presented increased EPIC sexual domain scores than the rest of the treatment groups (p < 0.01), but only statistically higher than RP for those in AS for longer than 2.5 years (Fig. 2a). Moreover, differences in hormonal scores between AS and the other treatment groups were only statistically significant among those with time longer than 2.5 years since treatment selection. No differences were found among the other domains (urinary irritative-obstructive and bowel). Figure 2b, with EPIC results stratified by age at HRQoL survey, shows a similar pattern of differences.

**Fig. 3:**
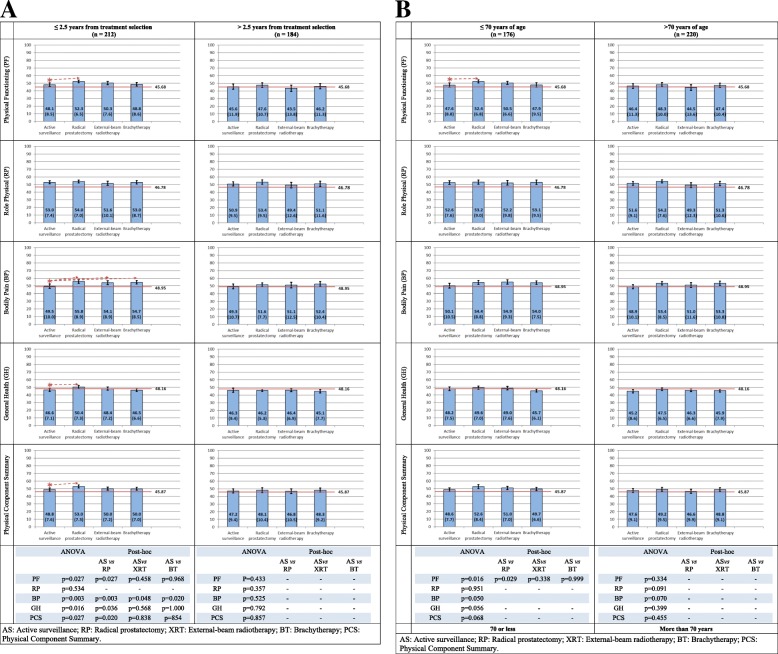
Physical health dimensions and Physical Component Summary of the SF-36 in patients with localized prostate cancer. Mean (SD) by treatment group. In red, US general population reference norm (men aged 65–74). **a** Stratified by elapsed time from treatment selection to HRQoL survey. **b** Stratified by age at HRQoL survey

Most of SF-36 physical health dimensions (Fig. 3a) presented statistically significant higher scores for RP groups than among patients in AS for < 2.5 years, while no differences were found among those with more than 2.5 years from treatment selection. Figure 3b, with results stratified by age, only shows a difference between AS and RP for physical functioning among patients < 70 years old.

**Fig. 4:**
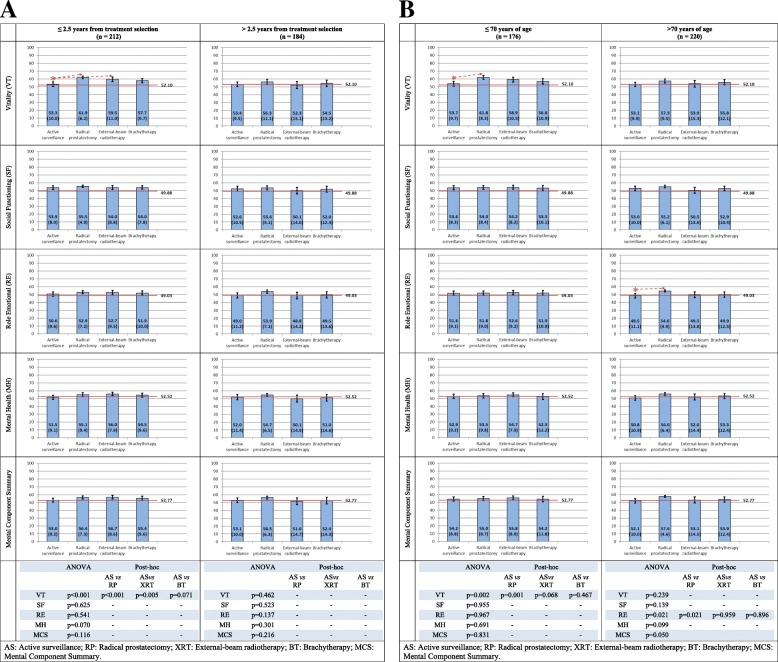
Mental health dimensions and Mental Component Summary of the SF-36 in patients with localized prostate cancer. Mean (SD) by treatment group. In red, US general population reference norm (men aged 65–74). **a** Stratified by elapsed time from treatment selection to HRQoL survey. **b** Stratified by age at HRQoL survey

No differences were found in SF-36 mental health dimensions among treatment groups (Fig. 4a nd b), except for lower (worse) scores in vitality among patients in AS for < 2.5 years or < 70 years of age (*p* = 0.001).

Figures 3 and 4 show that patients of all treatment groups reported similar or even better SF-36 scores than the general population reference norm (men aged 65–74).

## Discussion

Patients in the AS group presented better sexual outcomes than any active treatment at short term, difference which persisted only with the RP group beyond 2.5 years after treatment selection. The magnitude of the differences between AS and RP groups was large in both stratums of elapsed time to HRQoL survey (1.0 and 0.74 SD). This is explained by the well-known erectile dysfunction that RP may cause in some cases [3, 4]. Our good sexual results for patients in the AS program are similar to those reported in the USA observational study showing their statistically significant better sexual function than RP and XRT groups at 3rd year of follow-up [21]. Sexual dysfunction for patients in AS reported by ProtecT trial [4], the Australian cohort [19], and a USA study [20] could be partly explained by the ‘intention to treat’ analytical approach applied in these studies, and their worse results be caused by those patients who underwent radical prostatectomy or radiotherapy at some point during follow-up (14% [19], 19% [20], and 20% [22] at the 2nd year). A longitudinal study of predictors of sexual dysfunction in men on AS only identified age, diabetes and PSA as the factors independently associated [33].

In our study, patients in AS group reported higher urinary incontinence scores (better continence) compared to RP, with large differences regardless of time elapsed from treatment selection (0.99 and 0.74 SD). It may be explained by the loss of continence associated to surgery, although it usually improves over time [3, 4]. These results are in line with the lack of sexual and urinary continence side effects showed in a study that compared patients in AS with men without cancer after a prostate biopsy [34].

Regardless of the treatment applied, our results proved that physical and mental components of health are very similar to those reported by US general population, indicating a good health status in patients with favourable-risk PC. These results are consistent with those obtained in the ProtecT [4] and in a systematic review [9], which concluded that there was no major perturbation of HRQoL and psychological well-being in men undergoing AS in comparison to radical treatments. Nonetheless, patients in AS reported worse outcomes than other treatment groups at short term on physical and mental health domains: especially in bodily pain (0.46–0.63 SD) and vitality (0.55–0.77 SD). The magnitude of these differences was moderate. Pain-related effects of repeated prostate biopsies in the AS group could explain, partly, bodily pain differences. They can be also explained by differences at baseline between AS and RP groups: younger and healthier patients may tend to choose more active treatments like RP, with better subsequent recovery. In fact, differences between treatments in the physical health domains do not appear after stratifying by age.

Our main strengths are the HRQoL evaluation, which included two of the most widely used generic and disease-specific instruments at this moment, and the matching of patients in the AS program with the “Spanish Multicentric Study of Clinically Localized Prostate Cancer” cohort. Moreover, the cross-sectional design of the study clearly shows us the real HRQoL status of the patients remaining in the AS group, without contamination of those who switched to active treatments.

Our matching process reduced baseline differences, providing comparable groups. Statistically significant differences were found on mean age at diagnosis: The AS group’s mean age was intermediate between RP and XRT (see Table 1). Besides, patients in the AS group were more likely to be diagnosed in T2 stage in comparison to other treatments, which may surprise since AS is a strategy to delay or avoid curative treatment in localized PC [5–8], but we should bear in mind that AS patients are more likely to be defined by NRMI (nuclear magnetic resonance imaging), with consequent staging as T2. All groups were equivalent in terms of PSA and Gleason score.

The main limitation is that this is a cross-sectional study, not enabled to assess patients’ evolution or to avoid selection bias. Patients in AS for longer than 6 months assessed in this study were those who tolerate it well physically and mentally, and those who did not have progressive disease or symptoms resulting in termination of AS protocol. The study could also overestimate differences between AS and active treatments, especially because treatments in the interventional arms (RP, XRT and BT) were performed during the 2003–2005 period, so techniques and procedures have evolved. Nowadays, functional outcomes in these attempted curative treatments have progressed: slightly smaller declines in erectile function with robotic radical prostatectomy than with open technique [35, 36], and slightly better bowel results for IMRT than for older 3D conformal radiation therapy [37, 38]. Furthermore comorbidity, which could be a major factor in choice of therapy, was not collected for patients in AS. Finally, the SF-36 reference norms used were from the US general population. However, as the Spanish and US reference norms of SF-36 were very similar [25, 31] both of them are suitable and US reference norms facilitate international comparisons.

## Conclusions

In conclusion, considering treatment side effects and patients’ well being, AS can be a good therapeutic option due to the low impact caused on HRQoL. Moreover, patients undergoing AS do not seem to suffer major negative psychological effects in comparison to active treatments or general population. Our results provide a first approximation of AS patient-reported outcomes in HRQoL. However, longitudinal studies may be required to take into account HRQoL evolution over the years, in order to make clear recommendations showing the percentage of patients who remain in AS without radical treatment and their HRQoL at different follow-up times.

## Additional file

Additional file 1: **Table S1.** Comparison between matched and non-matched patients. (DOCX 19 kb)

## Abbreviations

AS: Active surveillance
HRQoL: Health-Related Quality of Life
LPC: Localized prostate cancer
RP: Radical prostatectomy
XRT: External-beam radiotherapy
BT: Brachytherapy
EPIC: Expanded Prostate Cancer Index Composite
SF-36: 36-Item Short Form Health Survey (SF-36)
PC: Prostate cancer
PSA: Prostate specific antigen
SF-36: The 36-Item Short Form Health Survey
SD: Standard deviation
ES: Effect size
NRMI: Nuclear magnetic resonance imaging
