# Supplementary material for: Health-related quality of life in men with prostate cancer undergoing active surveillance versus radical prostatectomy, external-beam radiotherapy, prostate brachytherapy and reference population: a cross-sectional study
Source: Health Qual Life Outcomes. 2019 Jan 14;17:11. doi: 10.1186/s12955-019-1082-4 (PMC6332524; doi:10.1186/s12955-019-1082-4)
Supplement: Supplementary file 1 — Table S1. Comparison between matched and non-matched patients. (DOCX 19 kb) [file 12955_2019_1082_MOESM1_ESM.docx]

**Supplementary table**. Comparison between matched and non-matched patients.

|  | Radical Prostatectomy | | | | External Radiotherapy | | | | Brachytherapy | | | |
| --- | --- | --- | --- | --- | --- | --- | --- | --- | --- | --- | --- | --- |
|  | Matched  sub-sample  (n=99) | Non-matched sub-sample  (n=93) | p-value | Matched sub-sample  (n=99) | | Non-matched sub-sample  (n=133) | p-value | Matched sub-sample  (n=99) | | Non-matched sub-sample  (n=220) | p-value |  |
| **Risk group** |  |  |  |  | |  |  |  | |  |  |  |
| Low risk | 74 (74.7%) | 16 (15.2%) | < 0.001 | 74 (74.7%) | | 34 (25.6%) | < 0.001 | 74 (74.7%) | | 209 (95.0%) | < 0.001 |  |
| Intermediate risk | 25 (25.3%) | 77 (73.3%) |  | 25 (25.3%) | | 61 (45.9%) |  | 25 (25.3%) | | 9 (4.1%) |  |  |
| High risk |  |  |  | 0 (0.0%) | | 38 (28.6%) |  | 0 (0.0%) | | 2 (0.9%) |  |  |
| **Age at treatment selection, mean (SD), years** | 65.1 (4.7) | 63.2 (6.1) | 0.014 | 70.4 (4.7) | | 69.4 (6.1) | 0.154 | 69.0 (4.6) | | 66.8 (6.9) | 0.001 |  |
| **PSA, mean (SD), ng/ml** | 7.3 (2.8) | 8.3 (3.7) | 0.035 | 7.7 (3.0) | | 11.5 (9.2) | < 0.001 | 7.6 (2.6) | | 6.7 (2.0) | 0.001 |  |
| Median [IQ range] | 6.9 [5.2 - 8.3] | 7.9 [5.4 – 10.0] |  | 7.6 [5.9 - 8.8] | | 8.0 [5.9 – 13.7] |  | 7.2 [5.9 - 8.8] | | 6.4 [5.3 – 7.8] |  |  |
| PSA ≤ 10 | 88 (89.8%) | 81 (75.7%) | 0.008 | 84 (84.8%) | | 90 (66.2%) | 0.001 | 85 (85.9%) | | 216 (97.3%) | < 0.001 |  |
| PSA > 10 | 10 (10.2%) | 26 (24.3%) |  | 15 (15.2%) | | 46 (33.8%) |  | 14 (14.1%) | | 6 (2.7%) |  |  |
| **Gleason score** |  |  |  |  | |  |  |  | |  |  |  |
| 2-5 | 12 (12.1%) | 4 (4.0%) | < 0.001 | 30 (30.3%) | | 24 (19.8%) | 0.001 | 28 (28.3%) | | 74 (33.6%) | 0.007 |  |
| 6 | 72 (72.7%) | 31 (31.0%) |  | 53 (53.5%) | | 51 (42.1%) |  | 64 (64.6%) | | 144 (65.5%) |  |  |
| 7 (3+4) | 15 (15.2%) | 65 (61.3%) |  | 16 (16.2%) | | 46 (33.8%) |  | 7 (7.1%) | | 2 (0.9%) |  |  |
| **Clinical stage** |  |  |  |  | |  |  |  | |  |  |  |
| T1a-T1c | 71 (71.7%) | 64 (61.0%) | 0.104 | 68 (68.7%) | | 56 (42.1%) | < 0.001 | 80 (80.3%) | | 180 (81.4%) | 0.892 |  |
| T2a-T2b | 28 (28.3%) | 41 (39.0%) |  | 31 (31.3%) | | 77 (57.9%) |  | 19 (19.2%) | | 41 (18.6%) |  |  |
| **Comorbidities** |  |  |  |  | |  |  |  | |  |  |  |
| Osteoarthritis or Rheumatism | 31 (43.1%) | 29 (44.6%) | 0.854 | 44 (48.9%) | | 61 (52.1%) | 0.643 | 40 (50.6%) | | 77 (38.7%) | 0.069 |  |
| High blood pressure | 26 (36.1%) | 28 (43.1%) | 0.405 | 33 (36.7%) | | 45 (38.5%) | 0.792 | 33 (41.8%) | | 81 (40.7%) | 0.870 |  |
| Depression or Mental disorders | 13 (18.1%) | 6 (9.2%) | 0.136 | 12 (13.3%) | | 20 (17.1%) | 0.458 | 17 (21.5%) | | 29 (14.6%) | 0.160 |  |
| Chronic respiratory diseases | 7 (9.7%) | 6 (9.2%) | 0.922 | 17 (18.9%) | | 25 (21.4%) | 0.660 | 16 (20.3%) | | 27 (13.6%) | 0.164 |  |
| Ischemic heart disease | 6 (8.3%) | 8 (12.3%) | 0.443 | 16 (17.8%) | | 19 (16.2%) | 0.770 | 11 (13.9%) | | 34 (17.1%) | 0.519 |  |
| Diabetes mellitus | 8 (11.1%) | 9 (13.8%) | 0.628 | 13 (14.4%) | | 17 (14.5%) | 0.986 | 10 (12.7%) | | 16 (8.0%) | 0.233 |  |
| Stroke | 2 (2.8%) | 2 (3.1%) | 0.917 | 3 (3.3%) | | 10 (8.5%) | 0.125 | 4 (5.1%) | | 12 (6.0%) | 0.755 |  |
